# Early ambulation after surgery in children with cerebral arteriovenous malformations: associated factors and care strategies

**DOI:** 10.3389/fneur.2026.1676483

**Published:** 2026-04-08

**Authors:** Xinjie Ren, Yu Zhang, Ran Ji, Wenzhao Wang, Tian Cao

**Affiliations:** Department of Neurosurgery, Children’s Hospital of Nanjing Medical University, Nanjing, Jiangsu Province, China

**Keywords:** ambulation, arteriovenous malformations, care, children, clinical, nursing, surgery

## Abstract

**Background:**

Early postoperative ambulation plays a crucial role in accelerating postoperative recovery in pediatric patients. This study aimed to analyze the current status and associated factors of early ambulation after surgery in children with cerebral arteriovenous malformations (AVMs).

**Methods:**

This study retrospectively enrolled pediatric patients with AVMs treated in our hospital between January 2022 and May 2025. Early postoperative ambulation was defined as the initiation of phased, progressive mobility within 12–48 h after surgery, based on pediatric neurosurgical ERAS principles to balance safety and recovery benefits. Clinical data of patients were collected and compared into two groups based on whether they underwent early postoperative ambulation.

**Results:**

A total of 156 pediatric patients with cerebral AVMs were enrolled, yielding an overall early postoperative ambulation rate of 39.74%. Correlation analyses identified statistically significant associations between early ambulation and five key variables: age (*r* = 0.613), admission Glasgow Coma Scale (GCS) score (*r* = 0.579), Spetzler-Martin classification (*ρ* = −0.616), surgical duration (*r* = −0.597), and parental education level (*ρ* = 0.586; all *p* < 0.05). Pearson’s correlation coefficients were used for continuous variables, while Spearman’s rank correlation coefficients were applied to ordinal categorical variables. Multivariate logistic regression analysis confirmed that children aged < 8 years (OR = 2.881, 95% CI: 1.820–3.633), those with admission GCS scores < 12 (OR = 2.900, 95% CI: 1.653–3.475), severe Spetzler-Martin classification (Grades III–V; OR = 3.021, 95% CI: 2.532–3.955), surgical duration ≥ 240 min (OR = 1.933, 95% CI: 1.251–2.604), and lower parental education level (OR = 1.681, 95% CI: 1.103–2.055) had significantly higher odds of not undergoing early ambulation (all *p* < 0.05).

**Conclusion:**

The rate of early postoperative ambulation in children with cerebral AVMs remains suboptimal, highlighting the need for targeted improvement strategies. Clinically, these findings underscore the importance of designing and implementing targeted nursing interventions that address these specific associated factors, with the goal of enhancing postoperative recovery in this vulnerable population.

## Introduction

Pediatric intracranial arteriovenous malformations (AVMs), a congenital vascular anomaly arising from aberrant cerebrovascular development during the embryonic stage, are characterized by direct arteriovenous shunts without an intervening capillary bed, forming abnormal vascular nidi that disrupt regional hemodynamics (1, 2). Although the prevalence of this condition in the pediatric population is merely 2–3 per 100,000, classifying it as a rare disease, its clinical impact is profoundly significant (3). Clinical data reveal that 65–85% of pediatric AVMs present with intracranial hemorrhage as the initial symptom, significantly higher than the 40–60% in adult patients (4–7). Currently, surgical resection remains the preferred treatment for Spetsler-Martin grade I-IV pediatric AVMs (8).

The quality of interventions during the postoperative rehabilitation phase directly determines the functional recovery of children. Early ambulation, a cornerstone of the Enhanced Recovery After Surgery (ERAS) protocol in neurosurgery, has been demonstrated to reduce the average length of hospital stay by 1.5 to 3 days. This benefit is mediated through several physiological mechanisms, including enhanced systemic circulation, decreased incidence of pulmonary complications, and prevention of deep vein thrombosis (9–11). However, the particularity of pediatric AVM patients poses multiple challenges to the implementation of early ambulation: age-related differences in compliance, weak compensatory capacity due to immature neurological development, and the direct impact of lesion location and the degree of surgical trauma on motor function may all alter the threshold of activity tolerance.

However, robust evidence specific to pediatric AVM surgery is lacking, leading to rehabilitation protocols often based on adult data or institutional experience rather than tailored evidence. This study aims to systematically identify factors associated with early ambulation in this unique population to inform optimized, evidence-based postoperative pathways.

## Methods

### Study design and ethics

This study adopted a retrospective cohort design. The protocol was reviewed and approved by the ethics committee of Children’s Hospital of Nanjing Medical University (approval number: 202509025–1). Written informed consent was obtained from the parents or legal guardians of all participating children, and the collected data were used exclusively for the purposes of this study.

### Participants

This study included pediatric patients with cerebral AVM who were diagnosed and treated in the Department of Neurosurgery of our hospital between January 2022 and May 2025. The inclusion criteria were set as follows: (1) Patients aged 0–18 years; (2) The diagnosis of cerebral AVM was confirmed by cranial imaging examinations (including digital subtraction angiography, magnetic resonance angiography, etc.), and the patients received surgical treatment in our hospital until discharge, with complete surgical records and pathological diagnosis data; (3) The parents or legal guardians of the patients voluntarily signed the informed consent form.

The exclusion criteria included: (1) Children with a history of hematological diseases (such as hemophilia, idiopathic thrombocytopenic purpura, etc.), or those with intracranial hemorrhage caused by other etiologies such as traumatic brain injury and aneurysm rupture; (2) Patients with incomplete key clinical data were excluded.

### Sample size and power

A post-hoc power analysis was performed using G Power 3.1 software (Faul, F., Erdfelder, E., Lang, A.-G., and Buchner, A., University of Düsseldorf, Germany). to verify the statistical power of our sample size, with the following parameters: effect size *w* = 0.30 (moderate effect), significance level *α* = 0.05, and number of predictors = 5 (age, GCS score, Spetzler-Martin classification, surgical duration, parental education level). The analysis indicated that a sample size of 156 achieved a statistical power of 0.92 (> 0.80). The analysis indicated that with our sample size of 156, the study had 92% power (> 80%, the conventional threshold) to detect a moderate effect size (*w* = 0.30) for the logistic regression model with five primary predictors at a significance level of *α* = 0.05.

### Definition and protocol of early ambulation

Early postoperative ambulation was defined as phased, progressive mobility initiated within 12–48 h after surgery, provided vital signs were stable and intracranial pressure was controlled. The protocol consisted of two phases:

Phase 1 (12–24 h): Bedside sitting (3–5 min/session, 2–3 sessions/day).

Phase 2 (24–48 h): Assisted standing (5–10 min/session, 2 sessions/day) or short-distance walking (10–20 steps/session, 1–2 sessions/day), as tolerated.

This protocol served as the standardized goal for all eligible children. The decision to initiate and advance through each phase was made jointly by the attending neurosurgeon and a specialized nurse, based on daily assessment of the child’s neurological status, vital signs, pain control, and overall tolerance. All activities were directly supervised, documented, and safety-assessed by trained pediatric neurosurgery nurses using the Pediatric Early Mobility Scale (PEMS) (12–14). A child was classified in the early ambulation group if they successfully completed at least Phase 1 (bedside sitting) within the 12–48-h window, as recorded in the nursing notes. Progression to Phase 2 was encouraged but not required for this classification. Parents were informed and encouraged to provide motivational support but were not responsible for initiating or timing the activities.

### Data collection

Relevant data of the children were collected from their medical records and nursing records, including the following items: whether the children ambulated within 12–48 h postoperatively, gender, age (years), body mass index (BMI, kg/m^2^), Glasgow Coma Scale (GCS) score at admission, feeding arteries, draining veins, lesion location, Spetsler-Martin classification (15), duration of surgery (minutes), parental age, and parental education level.

### Statistical analysis

All statistical analyses were performed using SPSS version 26.0 (IBM Corp., Armonk, NY, USA). Categorical variables are summarized as frequencies and percentages [n (%)], and continuous variables are reported as mean ± standard deviation (SD) or median with interquartile range based on distribution. Group comparisons were conducted as follows: categorical variables using the χ^2^ test or Fisher’s exact test where appropriate; normally distributed continuous variables using independent-samples t-test; and non-normally distributed continuous variables using the Mann–Whitney U test. Variables with *p* < 0.10 in univariate analyses were considered candidate variables for inclusion in the subsequent multivariable logistic regression model. The *p*-values in univariate comparisons are presented for descriptive screening and should be interpreted with awareness of multiple testing. Continuous variables (age and surgical duration) were dichotomized using clinically and statistically derived cut-offs: age (< 8 vs. ≥ 8 years) based on receiver operating characteristic (ROC) curve analysis, and surgical duration (≥ 240 vs. < 240 min) based on the 75th percentile of the sample distribution, to facilitate clinical interpretation. Correlation analyses between early ambulation and baseline characteristics were performed using Pearson’s correlation coefficient (r) for normally distributed continuous variables and Spearman’s rank correlation coefficient (*ρ*) for ordinal or non-normally distributed variables. Multicollinearity among predictors was assessed using variance inflation factors (VIFs), with all values < 2.0 indicating absence of significant collinearity. The discriminative ability of the logistic regression model was evaluated using the area under the ROC curve (AUC = 0.78), and model calibration was assessed with the Hosmer–Lemeshow goodness-of-fit test (*p* = 0.42). A two-tailed *p*-value < 0.05 was considered statistically significant.

## Results

A total of 156 pediatric patients with cerebral AVMs were included in this study, among whom 62 underwent early postoperative ambulation, resulting in an early ambulation rate of 39.7% (or approximately 40%). Patient characteristics are detailed in Table 1. Statistically significant differences were observed between the early ambulation group and the non-early ambulation group with respect to age (years), GCS score at admission, Spetsler-Martin classification, duration of surgery (minutes), and parental education level (all *p* < 0.05). Normality was confirmed for all continuous variables using the Shapiro–Wilk test (all *p* > 0.05). In contrast, no statistically significant differences were detected between the two groups in terms of gender, BMI, feeding arteries, draining veins, lesion location, or parental age (all *p* > 0.05).

**Table 1:** Characteristics of included children with cerebral arteriovenous malformations (*n* = 156).

Characteristic	Early ambulation group (*n* = 62)	No early ambulation group(*n* = 94)	t/F	*p*
Male/female	27/35	39/55	1.772	0.125
Age (years)	9.86 ± 2.14	7.02 ± 2.09	2.075	**0.032**
BMI (kg/m^2^)	14.58 ± 2.66	15.01 ± 2.58	3.044	0.106
GCS at admission	13.70 ± 1.55	9.80 ± 1.43	2.436	**0.018**
Feeding arteries			1.904	0.133
Anterior circulation	34(54.84%)	52(55.32%)		
Posterior circulation	28(45.16%)	42(44.68%)		
Draining veins			1.877	0.160
Superficial venous drainage	48(77.42%)	72(76.60%)		
Deep venous drainage	14(22.58%)	22(23.40%)		
Lesion location			1.235	0.213
Supratentorial	45(72.58%)	72(76.60%)		
Infratentorial	12(19.35%)	17(18.08%)		
Thalamus, basal ganglia, corpus callosum	5(8.06%)	5(5.32%)		
Spetsler-Martin classification			1.589	**0.002**
I	10(16.13%)	9(9.57%)		
II	30(48.39%)	27(28.72%)		
III	18(29.03%)	43(45.74%)		
IV	3(4.84%)	10(10.64%)		
V	1(1.61%)	5(5.32%)		
Duration of surgery (min)	206.21 ± 42.68	270.15 ± 50.44	35.004	**0.028**
Parental age	38.28 ± 6.40	37.92 ± 5.50	6.516	0.104
Parental education level			1.490	**0.032**
Primary school or below	2(3.22%)	18(19.15%)		
Junior high school	4(6.45%)	23(24.47%)		
Senior high school	24(38.71%)	38(40.43%)		
Associate degree	10(16.14%)	10(10.64%)		
Bachelor’s degree or above	22(35.48%)	5(5.32%)		

Data are presented as n (%) or mean ± standard deviation. BMI, body mass index; GCS, Glasgow Coma Scale. A Shapiro–Wilk test confirmed normality for all continuous variables (all p > 0.05). *p*-values < 0.05 are indicated in bold.

As presented in Table 2, correlation analyses revealed significant associations between early postoperative ambulation in children with cerebral AVMs and the following variables: age (*r* = 0.613), GCS score at admission (*r* = 0.579), Spetzler-Martin classification (*ρ* = −0.616), duration of surgery (*r* = −0.597), and parental education level (*ρ* = 0.586) (all *p* < 0.05). Pearson’s correlation coefficient (r) was used for continuous variables (age, GCS score, duration of surgery, BMI, parental age), while Spearman’s rank correlation coefficient (ρ) was applied for ordinal categorical variables (Spetzler-Martin classification, parental education level, gender, feeding arteries, draining veins, lesion location), in accordance with the variable types.

**Table 2:** Correlation analysis of early ambulation and characteristics of children with cerebral arteriovenous malformations.

Variables	Correlation coefficient (*r*/*ρ*)	*p*
Gender	0.121 (ρ)	0.114
Age (y)	**0.613 (r)**	**0.030**
BMI (kg/m^2^)	0.120 (r)	0.098
GCS at admission	**0.579 (r)**	**0.015**
Feeding arteries	0.105 (*ρ*)	0.113
Draining veins	0.116 (ρ)	0.121
Lesion location	0.174 (ρ)	0.069
Spetzler-Martin classification	**−0.616 (ρ)**	**0.007**
Duration of surgery (min)	**−0.597 (r)**	**0.021**
Parental age	0.163 (r)	0.115
Parental education level	**0.586 (ρ)**	**0.040**

*r*, Pearson’s correlation coefficient; ρ, Spearman’s rank correlation coefficient. Statistically significant correlations (*p* < 0.05) are indicated in bold.

The variable coding scheme is shown in Table 3. Critical revisions were made to the coding of the outcome variable (early ambulation) and the ordinal Spetzler-Martin classification to ensure logical consistency: the outcome variable was recoded as 1 = yes and 0 = no, and Spetzler-Martin grades were assigned sequential values from 1 (Grade I) to 5 (Grade V). As outlined in Table 4, multivariate logistic regression analysis identified that children with age < 8 years (OR = 2.881, 95% CI: 1.820 ~ 3.633), GCS score < 12 at admission (OR = 2.900, 95% CI: 1.653 ~ 3.475), severe Spetzler-Martin classification (Grades III–V) (OR = 3.021, 95% CI: 2.532 ~ 3.955), surgical duration ≥ 240 min (OR = 1.933, 95% CI: 1.251 ~ 2.604), and lower parental education level (OR = 1.681, 95% CI: 1.103 ~ 2.055) had significantly higher odds of not undergoing early ambulation (all *p* < 0.05).

**Table 3:** Variable coding scheme for multivariate logistic regression analysis.

Variable	Description	Coding scheme
Early ambulation (Y)	Postoperative mobility outcome	1 = Yes (early ambulation), 0 = No (no early ambulation)
Age	Chronological age at surgery (years)	1 = < 8 years, 2 = ≥ 8 years
GCS at admission	Glasgow Coma Scale score upon admission	1 = < 12, 2 = ≥ 12
Spetzler-Martin classification	Anatomic grading of AVM severity	1 = Grade I, 2 = Grade II, 3 = Grade III, 4 = Grade IV, 5 = Grade V (entered as ordinal variable)
Duration of surgery	Total operative time (minutes)	1 = ≥ 240 min, 2 = < 240 min
Parental education level	Highest educational attainment of parents	1 = Primary school or below, 2 = Junior high school, 3 = Senior high school, 4 = Associate degree, 5 = Bachelor’s degree or above (entered as ordinal variable)

All variables were entered into the logistic regression model using the coding scheme above. Reference categories are indicated by the higher numerical values for dichotomous variables.

**Table 4:** Multivariate logistic regression analysis of factors associated with absence of early postoperative ambulation.

Risk factor	*β*	SE	OR	95% CI	*p*-value
Age < 8 years (vs. ≥ 8 years)	0.815	0.140	2.881	1.820–3.633	0.021
Admission GCS < 12 (vs. ≥ 12)	0.662	0.115	2.900	1.653–3.475	0.014
Severe Spetzler-Martin grade (III–V vs. I–II)	0.893	0.267	3.021	2.532–3.955	0.005
Surgical duration ≥ 240 min (vs. < 240 min)	0.606	0.182	1.933	1.251–2.604	0.031
Lower parental education level (per unit decrease)^1^	0.927	0.134	1.681	1.103–2.055	0.046

*β*, regression coefficient; SE, standard error; OR, odds ratio; CI, confidence interval; GCS, Glasgow Coma Scale.

^1^Parental education level was entered as an ordinal variable (1 = primary school or below to 5 = bachelor’s degree or above). The OR reflects increased odds of not ambulating early for each one-level decrease in educational attainment.

Model performance: Area under the ROC curve (AUC) = 0.78, indicating acceptable discriminative ability. Hosmer–Lemeshow goodness-of-fit test: χ^2^ = 8.24, *p* = 0.42, suggesting good model calibration. All variance inflation factors (VIFs) were < 2.0, indicating no significant multicollinearity among predictors.

## Discussion

The early postoperative ambulation rate in our pediatric AVM cohort was 39.7%, which falls below the 50–60% range typically reported in adult neurosurgical populations (16, 17). This rate is comparable to, though slightly lower than, the 42.3% recently documented in pediatric brain tumor resection (18), a discrepancy that may reflect the greater vascular complexity and intraoperative trauma inherent to AVM obliteration. Conversely, our rate exceeds the 31.5% reported in pediatric traumatic brain injury patients (19), likely due to the more profound acute neurological compromise in that population. Pediatric AVMs are known to present with more aggressive phenotypes and higher surgical complexity than their adult counterparts (20, 21), suggesting that even this modest ambulation rate represents a meaningful achievement in a uniquely challenging cohort. Collectively, these comparisons underscore that early ambulation in pediatric AVM patients is constrained by the convergence of disease-specific factors, immature physiological reserve, and perioperative safety imperatives.

At our institution, all pediatric AVM cases are managed through a dedicated neurovascular multidisciplinary team (MDT) comprising neurosurgeons, interventional neuroradiologists, neurologists, and neuroanesthesiologists. Treatment decisions are individualized based on lesion characteristics, patient age, clinical presentation, and family preferences. While surgical resection remains the preferred approach for Spetzler-Martin Grade I–IV AVMs, endovascular embolization (as standalone therapy for select deep-seated lesions or as preoperative adjunct) and stereotactic radiosurgery (for small, deep, or residual lesions) are also offered within this multimodal framework. All patients in this cohort underwent elective surgery following initial stabilization; those presenting with acute hemorrhage or instability were managed conservatively until clinical optimization. The admission GCS score, a key indicator of preoperative neurological status, was significantly associated with early ambulation outcomes in our analysis.

Age < 8 years emerged as a significant determinant of early ambulation, reflecting the developmental limitations of younger children. Immature neuromuscular coordination, poor tolerance to postural changes, and postoperative symptoms such as dizziness and fatigue collectively diminish activity willingness (22). Additionally, younger children struggle to articulate discomfort, complicating risk assessment during mobilization (23). These findings support age-stratified nursing interventions: for children aged 3–8 years, gamified rehabilitation programs (e.g., cartoon-based sitting encouragement) and modified activity tolerance assessments (e.g., toy-grasping duration) may prove beneficial. For infants under 3 years, passive activities such as limb massage and positional changes should precede active mobilization, with continuous vital sign monitoring.

Lower admission GCS scores and higher Spetzler-Martin grades were associated with reduced ambulation rates, directly reflecting the impact of lesion severity on neurological function. Higher-grade lesions frequently involve eloquent areas (e.g., motor cortex, corticospinal tracts) or are complicated by hemorrhage, resulting in impaired consciousness, neurological deficits, and objectively limited activity capacity (24–26). The Spetzler-Martin classification indirectly captures these risks through its incorporation of lesion size, eloquence, and venous drainage pattern—factors that may contribute to postoperative motor compromise. Accordingly, dynamic rehabilitation pathways stratified by neurological status are warranted: for children with GCS 13–15 and Grade I–II lesions, sitting training may commence at 24 h postoperatively; for those with GCS 9–12 or Grade III lesions, bedside passive activities should proceed only after confirming intracranial pressure stability (ICP < 20 mmHg for 6 consecutive hours); for children with GCS < 9, active mobilization should be deferred until consciousness improves, with emphasis on pressure ulcer and deep vein thrombosis prevention during this period (27).

Surgical duration ≥ 240 min independently predicted lower likelihood of early ambulation through three interconnected mechanisms. First, prolonged anesthesia exposure (≥ 4 h) delays arousal and motor recovery in pediatric patients due to immature drug metabolism, directly impeding early mobility initiation (28). Second, extended surgical manipulation amplifies systemic inflammatory responses (e.g., interleukin-6, tumor necrosis factor-*α*) (29), inducing postoperative fatigue, myalgia, and neuroinflammation that diminish activity tolerance. Third, longer procedures correlate with greater tissue trauma and elevated pain scores (≥ 4 on FLACC scale), which deter mobilization (30). In response, targeted perioperative strategies should include: optimized preoperative fasting (clear liquids up to 2 h preoperatively) to reduce nausea and fatigue; intraoperative temperature maintenance (36–37 °C) to mitigate stress responses; and stepped postoperative pain management ensuring pain scores ≤ 3 before activity initiation, with near-infrared spectroscopy monitoring to safeguard against intracranial pressure fluctuations during mobilization (31).

Lower parental education level emerged as a social determinant of early ambulation, reflecting health literacy disparities that influence rehabilitation compliance. Parents with limited education often inadequately appreciate the benefits of postoperative activity and may refuse participation due to unfounded concerns about wound dehiscence or disease exacerbation (32, 33). Addressing this requires targeted educational interventions: graphic-aided materials (e.g., comic-style activity guides) to reduce comprehension barriers; collaborative “healthcare provider–parent” decision-making with preoperative clarification of phased activity goals; and peer support groups where parents of successfully rehabilitated children share experiences to build confidence (34). Digital tools such as WeChat mini-programs offering video demonstrations and timed reminders may further enhance implementation capacity (35).

## Limitations

Several limitations merit consideration. First, the single-center retrospective design precludes causal inference and may introduce unmeasured confounding, including variations in postoperative analgesia, nursing protocols, or transient clinical complications not captured in medical records. Institutional practice patterns may also limit generalizability, underscoring the need for multi-center validation. Second, while our sample size (*n* = 156) achieved adequate power for primary analyses (92% power to detect moderate effects), it may have been insufficient to identify more subtle associations. Third, dichotomization of continuous variables (age, surgical duration), though clinically practical, reduces data granularity and may obscure nuanced relationships. Fourth, the absence of longitudinal follow-up precludes assessment of early ambulation’s impact on long-term functional recovery, neurocognitive development, or quality of life. Finally, regarding preoperative status, all patients underwent elective surgery after stabilization; the admission GCS score served as the primary indicator of neurological condition, while the Spetzler-Martin grade indirectly captured risks associated with eloquent location or hemorrhagic presentation. Future prospective, multi-center studies should incorporate serial postoperative assessments, detailed pain trajectories, psychosocial variables (e.g., parental health literacy, caregiver burden), and extended follow-up to evaluate sustained effects of early mobilization on long-term outcomes in this vulnerable population.

## Conclusion

In this cohort, early postoperative ambulation was achieved in 39.7% of pediatric patients with cerebral arteriovenous malformations and was significantly associated with several factors: older age, higher admission GCS score, lower Spetzler-Martin grade, shorter surgical duration, and higher parental education level. These include both non-modifiable patient characteristics (age, admission neurological status, AVM grade) and potentially modifiable clinical (surgical duration) and social (parental education) factors that clinicians should consider when planning postoperative rehabilitation. Future prospective research should focus on developing and validating tailored, multi-faceted intervention pathways that address these factors, with the goal of safely improving early mobility rates in this vulnerable population.

## Data Availability

The original contributions presented in the study are included in the article/supplementary material, further inquiries can be directed to the corresponding author/s.
